# Deep-Learning-Based Reconstruction of Single-Breath-Hold 3 mm HASTE Improves Abdominal Image Quality and Reduces Acquisition Time: A Quantitative Analysis

**DOI:** 10.3390/curroncol32010030

**Published:** 2025-01-03

**Authors:** Felix Kubicka, Qinxuan Tan, Tom Meyer, Dominik Nickel, Elisabeth Weiland, Moritz Wagner, Stephan Rodrigo Marticorena Garcia

**Affiliations:** 1 Department of Radiology, Charité—Universitätsmedizin Berlin, Freie Universität Berlin, Humboldt-Universität zu Berlin and Berlin Institute of Health, Charitéplatz 1, 10117 Berlin, Germanymoritz.wagner@charite.de (M.W.); stephan.marticorena-garcia@charite.de (S.R.M.G.); 2 MR Applications Predevelopment, Siemens Healthcare GmbH, Allee am Roethelheimpark 2, 91052 Erlangen, Germanyelisabeth.weiland@siemens-healthineers.com (E.W.)

**Keywords:** MRI, deep learning, thin slice, HASTE, abdomen, high resolution

## Abstract

**Purpose:** Breath-hold T2-weighted half-Fourier acquisition single-shot turbo spin echo (HASTE) magnetic resonance imaging (MRI) of the upper abdomen with a slice thickness below 5 mm suffers from high image noise and blurring. The purpose of this prospective study was to improve image quality and accelerate imaging acquisition by using single-breath-hold T2-weighted HASTE with deep learning (DL) reconstruction (DL-HASTE) with a 3 mm slice thickness. **Method:** MRI of the upper abdomen with DL-HASTE was performed in 35 participants (5 healthy volunteers and 30 patients) at 3 Tesla. In a subgroup of five healthy participants, signal-to-noise ratio (SNR) analysis was used after DL reconstruction to identify the smallest possible layer thickness (1, 2, 3, 4, 5 mm). DL-HASTE was acquired with a 3 mm slice thickness (DL-HASTE-3 mm) in 30 patients and compared with 5 mm DL-HASTE (DL-HASTE-5 mm) and with standard HASTE (standard-HASTE-5 mm). Image quality and motion artifacts were assessed quantitatively using Laplacian variance and semi-quantitatively by two radiologists using five-point Likert scales. **Results:** In the five healthy participants, DL-HASTE-3 mm was identified as the optimal slice (SNR 23.227 ± 3.901). Both DL-HASTE-3 mm and DL-HASTE-5 mm were assigned significantly higher overall image quality scores than standard-HASTE-5 mm (Laplacian variance, both *p* < 0.001; Likert scale, *p* < 0.001). Compared with DL-HASTE-5 mm (1.10 × 10^−5^ ± 6.93 × 10^−6^), DL-HASTE-3 mm (1.56 × 10^−5^ ± 8.69 × 10^−6^) provided a significantly higher SNR Laplacian variance (*p* < 0.001) and sharpness sub-scores for the intestinal tract, adrenal glands, and small anatomic structures (bile ducts, pancreatic ducts, and vessels; *p* < 0.05). Lesion detectability was rated excellent for both DL-HASTE-3 mm and DL-HASTE-5 mm (both: 5 [IQR4–5]) and was assigned higher scores than standard-HASTE-5 mm (4 [IQR4–5]; *p* < 0.001). DL-HASTE reduced the acquisition time by 63–69% compared with standard-HASTE-5 mm (*p* < 0.001). **Conclusions**: DL-HASTE is a robust abdominal MRI technique that improves image quality while at the same time reducing acquisition time compared with the routine clinical HASTE sequence. Using ultra-thin DL-HASTE-3 mm results in an even greater improvement with a similar SNR.

## 1. Introduction

Magnetic resonance imaging (MRI) of the abdomen provides high soft tissue contrast but at the expense of long examination times and susceptibility to motion artifacts. Over the last few decades, various techniques have been developed to improve the image quality of T2-weighted (T2w) MR sequences by reducing respiratory or motion artifacts while at the same time reducing the examination time. These techniques include half-Fourier acquisition single-shot turbo spin echo (HASTE) sequencing [1], respiratory-triggered acquisition [2,3], and parallel imaging [4], which have all become part of clinical routine.

As a single-shot sequence, HASTE is the most motion-robust T2w sequence in abdominal imaging and can be acquired during breath-holds [5,6]. It is recommended that an abdominal imaging protocol should include an axial HASTE sequence with a slice thickness of at most 4–5 mm [7]. However, even with 4–5 mm thick slices, small anatomic or pathologic structures may not be detected, or the course of small structures may not be followed continuously. Acquisition of breath-hold HASTE of the upper abdomen with thinner slices is inhibited by an insufficient signal-to-noise ratio (SNR), which decreases with a greater slice thickness. Therefore, breath-hold thin-slice HASTE is currently only performed for magnetic resonance cholangiopancreatography (MRCP) with heavy T2w and limited anatomic coverage [8].

Algorithms with trainable components, referred to as deep learning (DL), provide a potential solution for performing thin-slice breath-hold HASTE with larger anatomic coverage. DL allows for image reconstruction with a high image quality from undersampled and noisy k-space data. DL is very versatile and can be used to shorten image acquisition and to improve image quality [9,10,11,12,13,14]. Previous studies have shown that 4–5 mm HASTE of the upper abdomen is feasible with DL-based reconstruction, allowing for single-breath-hold acquisition with a high image quality [15,16,17,18]. For example, Liu et al. [19] recently showed that DL-HASTE enables a 4 mm slice with clinical acceptable image quality for detecting pancreatic lesions, and Gassenmaier et al. [20] reported that 3 mm slice turbo spin echo (TSE) T2w sequencing of the prostate provided superior image quality compared to conventional T2-weighted imaging sequencing without increasing the acquisition time. For upper abdominal imaging, Tajima et al. demonstrated the feasibility of 3 mm DL-based T2w imaging at 1.5 T MRI by using several breath-holds [21]. We hypothesize that the HASTE image quality can be improved by using DL-based reconstruction (3 mm and 5 mm voxel slice thickness) without any loss of the SNR due to a concurrently reduced acquisition time.

The purpose of this study was to identify the lowest layer thickness after DL reconstruction when reaching an SNR plateau for the best-possible image sharpness and to improve the sharpness of abdominal imaging with a special focus on small anatomic structures in a larger dataset.

## 2. Materials and Methods

This prospective study complied with the Declaration of Helsinki and was approved by the internal review board of Charité—Universitätsmedizin Berlin, Germany (EA1/344/21). All participants gave written informed consent. Siemens Healthcare (in Erlangen, Bavaria, Germany) provided financial and technical support for the conduction of thing study. The authors had sole control of the data and information submitted for publication.

### 2.1. Study Population

Thirty patients and five healthy volunteers were investigated. We initially enrolled 31 consecutive patients who underwent clinical indicated upper abdominal MRI between March and October 2022. All patients gave informed consent to participate in this study. One patient was excluded from the analysis because of severe ascites and artifacts related to overall strong magnetic field inhomogeneities. The final analysis was conducted in 30 patients (mean age, 64.8 +/− 14.37 years; range 24–83 years; 15 females; suspected liver lesions, *n* = 8; pancreatic lesions, *n* = 12; kidney lesions, *n* = 10). In addition, five healthy participants without any history of abdominal disease were enrolled in an independent subgroup for MRI with HASTE acquisition at five different slice thicknesses.

### 2.2. MRI System and Acquisition Parameters

All examinations were performed on the same clinical 3 Tesla MRI system (MAGNETOM Vida; Siemens Healthcare, Erlangen, Bavaria, Germany) with subjects in the supine position using an 18-channel body/spine array coil. The imaging parameters are summarized in Table 1.

### 2.3. HASTE with DL Reconstruction

The HASTE sequence with deep learning (DL)-based reconstruction used in our study is a research tool consisting of a modified pulse sequence and reconstruction algorithm, as previously described in [13,15,16,22]. The sequence acquires k-space data using a regular sampling scheme, as known from parallel imaging, with separate acquisition of calibration data for generating coil sensitivity maps. To reduce crosstalk in acquisitions with a short repetition time (TR), the slice increment between consecutively acquired lines is increased to four. Furthermore, variable flip-angle evolution is supported for refocusing pulses in the echo train. The DL reconstruction is based on a variational network that receives undersampled k-space data as well as precalculated coil sensitivity maps as inputs and which generates the final images in an iterative process that alternates between a parallel imaging-based data consistency update and hierarchical neural network-based image enhancement. Training was performed offline in a supervised manner using about 10,000 slices acquired in the volunteers who gave written informed consent to this use of their imaging data in accordance with local IRB guidelines. The obtained network parameters were then exported and provided by Siemens Healthineers (in Erlangen, Bavaria, Germany) for prospective use in the reconstruction pipeline of the scanner.

### 2.4. Analysis of Signal-to-Noise Ratio (SNR)

For SNR analysis, five DL-HASTE sequences with slice thicknesses of 1, 2, 3, 4, and 5 mm were consecutively acquired in five healthy participants. The SNR at different resolutions was calculated as the mean divided by the standard deviation in a manually drawn homogeneous region of interest (ROI) within the spleen. The best tradeoff between the SNR and resolution was determined visually using an approximation of a plateau on the graph. The voxel slice thickness with the lowest SNR on the plateau was considered the best.

### 2.5. Quantitative Analysis of MR Image Quality

For the quantification of image sharpness, 30 patient images were registered onto the standard HASTE sequence using rigid registration with the elastix toolbox [23] and normalized to a range between 0 and 1. Laplacian variance was used as the sharpness metric [24,25].

### 2.6. Qualitative Assessment of MR Image Quality

Image data of 30 patients were presented in random order to two blinded radiologists (with 4 and 2 years of clinical experience in abdominal MRI) who performed the image analysis independently. Each observer simultaneously evaluated and compared three sequences per patient (standard-HASTE-5 mm, DL-HASTE-5 mm, DL-HASTE-3 mm). The two readers evaluated the following criteria: overall image quality; delineation of abdominal organs (liver: liver hilum plane, spleen: splenic hilum plane, kidney: renal hilum plane, and the entire pancreas); detailed anatomic structures (intestinal walls, common hepatic duct, cystic duct, common bile duct, main pancreatic duct, adrenals, renal parenchyma, renal sinus); conspicuity of major abdominal vessels (abdominal aorta, portal vein, celiac trunk, splenic vessels, renal vessels, and superior mesenteric artery [SMA]); and presence of breathing-related motion artifacts. For each of these features, a 5-point Likert score was assigned according to the definitions compiled in Table 2.

### 2.7. Lesion Detection

The same two readers independently reported the number of liver lesions, maximum lesion diameter, and lesion characteristics (solid, simple cyst, septated cyst). Only lesions measuring at least 5 mm in diameter were included. In addition, lesion detectability was evaluated using the same 5-point Likert scale (Table 2). Reading scores were considered sufficient when reaching a score of four or higher [15,18].

### 2.8. Statistical Analysis

Non-parametric variables are presented as medians and interquartile ranges (IQR) of the mean scores of two observers. We applied the paired Wilcoxon signed-rank test to compare image quality between DL-based reconstruction and routine clinical reconstruction. The Wilcoxon signed-rank test for continuous variables was used to compare acquisition times and liver lesion sizes for each sequence after a non-normal data distribution was confirmed by the Shapiro–Wilk test. For each sequence, we evaluated consistency in reader scores on all assessed parameters using intraclass correlation coefficient (ICC) analysis, where <0.4 = poor agreement; 0.4–0.59 = fair agreement; 0.6–0.74 = good agreement; and 0.75–1 = excellent agreement [18]. *p*-values less than 0.05 were considered to indicate a statistically significant difference. All statistical analyses were performed using SPSS version 29 (IBM Corp, Armonk, NY, USA).

## 3. Results

### 3.1. Signal-to-Noise Ratio (SNR) Analysis

**Figure 1:**
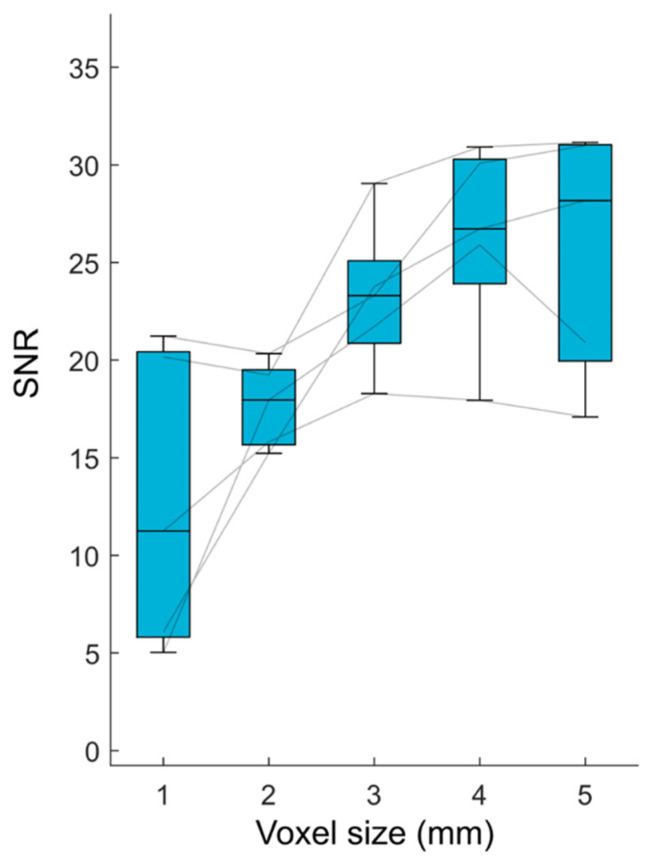
Analysis of best tradeoff between signal-to-noise ratio (SNR) and resolution. Visually, a relative plateau is recognizable between 3 and 5 mm voxel slice thicknesses. Therefore, a voxel slice thickness of 3 mm was considered as the best ratio of the lowest SNR on the plateau.

An SNR plateau was observed for DL-HASTE with voxel slice thicknesses of 3–5 mm (SNR, DL-HASTE 1 mm, 12.749 ± 7.639; 2 mm, 17.714 ± 2.182; 3 mm, 23.227 ± 3.901; 4 mm, 26.314 ± 5.144; 5 mm, 25.664 ± 6.343). A voxel slice thickness of 3 mm was considered optimal for the ratio between the highest SNR on the plateau and the slice thickness, see Figure 1.

### 3.2. Interobserver Agreement

Interobserver agreement between the two readers for all assessed parameters was good-to-excellent for standard-HASTE-5 mm and DL-HASTE (standard-HASTE-5 mm: 0.76; range 0.73–0.80; DL-HASTE-5 mm: 0.77; range 0.73–0.80; DL-HASTE-3 mm: 0.85; range 0.83–0.88). In the following section, the mean image quality scores of the two observers are reported.

### 3.3. Quantitative Assessment of Overall Image Quality

**Figure 2:**
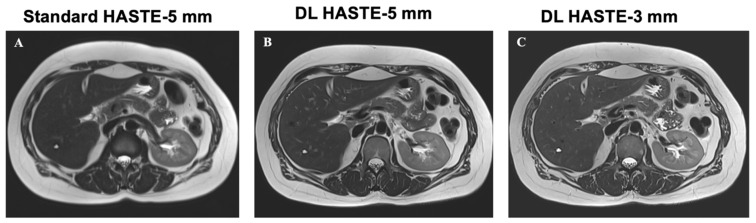
Image quality of clinical standard-HASTE-5 mm (**A**), DL-HASTE-5 mm (**B**), and DL-HASTE-3 mm (**C**). Image quality scores were higher for DL-HASTE ((**B**,**C**), excellent image quality) compared with standard-HASTE-5 mm ((**A**), good image quality).

**Figure 3:**
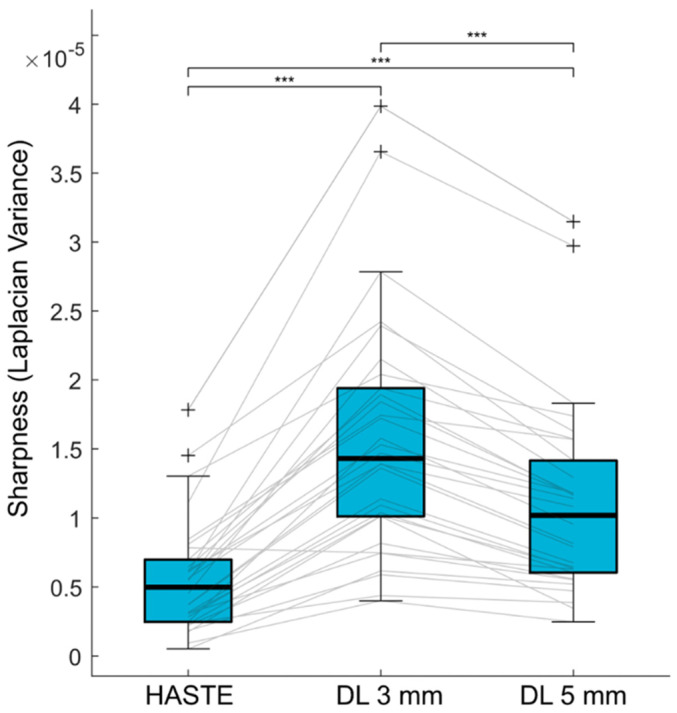
Quantitative analysis revealed higher sharpness for DL-HASTE-3 mm (1.56 × 10^−5^ ± 8.69 × 10^−6^) compared with DL-HASTE-5 mm (1.10 × 10^−5^ ± 6.93 × 10^−6^, ***: *p* < 0.001) and standard-HASTE-5 mm (5.59 × 10^−6^ ± 4.12 × 10^−6^, *p* < 0.001). Sharpness of DL-HASTE-5 mm was again higher than that of standard-HASTE-5 mm (*p* < 0.001).

Representative examples of standard-HASTE-5 mm, DL-HASTE-5 mm, and DL-HASTE-3 mm are presented in Figure 2. The sharpness was higher in DL-HASTE-3 mm (1.56 × 10^−5^ ± 8.69 × 10^−6^) compared to DL-HASTE-5 mm (1.10 × 10^−5^ ± 6.93 × 10^−6^, *p* = 6.80 × 10^−10^) and standard-HASTE-5 mm (5.59 × 10^−6^ ± 4.12 × 10^−6^, *p* = 1.23 × 10^−9^). Comparison of the standard and deep-learning-based reconstructions at the same slice thickness revealed a higher sharpness for DL-HASTE-5 mm than standard-HASTE-5 mm (*p* = 3.88 × 10^−7^), see Figure 3.

### 3.4. Semi-Quantitative Assessment of Image Quality

The overall image quality was rated superior in DL-HASTE-5 mm (5 [IQR, 4–5]) and DL-HASTE-3 mm (5 [IQR, 4–5]) compared with clinical standard-HASTE-5 mm (4 [IQR, 4–4], *p* < 0.001); no significant difference was found between DL-HASTE-5 mm and DL-HASTE-3 mm (*p* = 0.66). Furthermore, the score for motion artifacts was also better for DL-HASTE, with a median of 5 (IQR, 4–5) versus 4 (IQR, 4–5) for standard-HASTE (*p* < 0.005), but it did not differ between DL-HASTE-5 mm and DL-HASTE-3 mm (*p* = 0.64), see Table 3.

**Figure 4:**
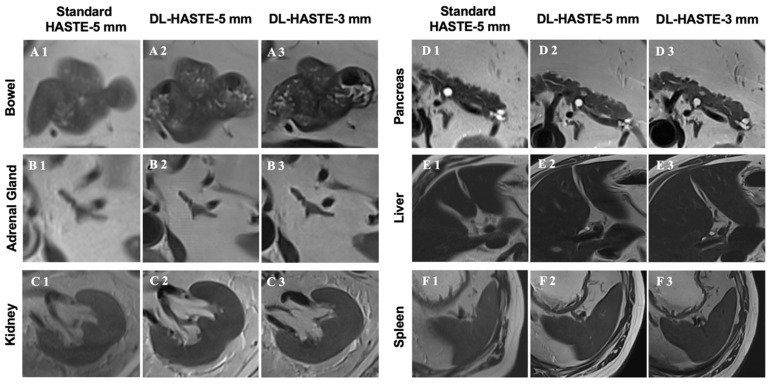
Sharpness of abdominal organ margins was compared between standard-HASTE-5 mm (**A1**,**B1**,**C1**,**D1**,**E1**,**F1**), DL-HASTE-5 mm (**A2**,**B2**,**C2**,**D2**,**E2**,**F2**), and DL-HASTE-3 mm (**A3**,**B3**,**C3**,**D3**,**E3**,**F3**). Note the improved sharpness of the bowel and adrenal gland on DL-HASTE-3 mm compared with DL-HASTE-5 mm (**A3** vs. **A2**, **B3** vs. **B2**). DL-HASTE occasionally showed tiny zebra-striped artifacts that did not interfere with the diagnostic image quality (**B2**,**D2**,**D3**).

**Figure 5:**
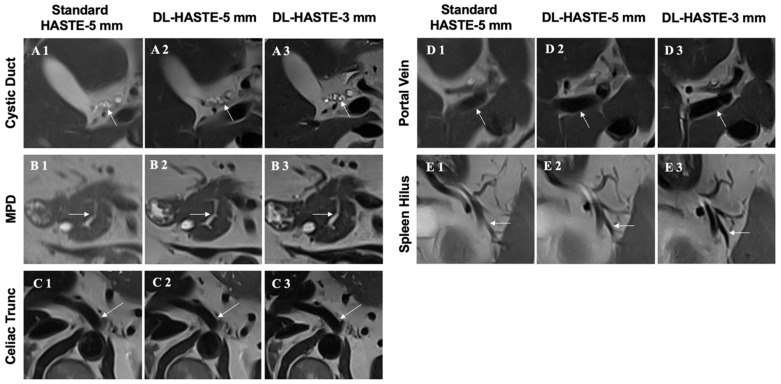
Conspicuity of small abdominal anatomic structures such as cystic duct ((**A**), arrows), main pancreatic duct ((**B**), arrows), celiac trunk ((**C**), arrows), portal vein ((**D**), arrows), and splenic hilum vessels ((**E**), arrows). DL-HASTE-3 mm (**A1**,**B1**,**C1**,**D1**,**E1**) achieved higher image quality rating scores than DL-HASTE-5 mm (**A2**,**B2**,**C2**,**D2**,**E2**) and clinical standard-HASTE-5 mm (**A3**,**B3**,**C3**,**D3**,**E3**). MPD = main pancreatic duct.

The detailed image quality of all anatomic regions was rated higher for DL-HASTE-5 mm and DL-HASTE-3 mm compared with standard-HASTE-5 mm (*p* < 0.003), see Figure 4 and Figure 5). DL-HASTE-3 mm was superior to DL-HASTE-5 mm in terms of overall sharpness, delineation of intestinal walls and adrenal glands, and visibility of small anatomic structures like the common hepatic duct, cystic duct, common bile duct, and main pancreatic duct, sharpness of the portal vein, SMA, celiac trunk, and splenic and renal vessels (all *p* < 0.001). No differences in edge acuity were observed for the liver, spleen, and abdominal aorta (*p* > 0.08). Detailed results are provided in Table 3.

DL-HASTE infrequently showed tiny zebra-striped artifacts that did not interfere with the diagnostic image quality, see Figure 4(B2,D2,D3).

### 3.5. Lesion Detection

**Figure 6:**
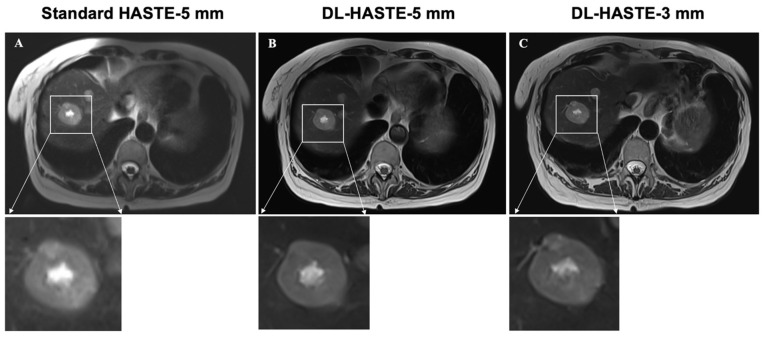
Delineation of solid liver lesion on clinical standard-HASTE-5 mm (**A**), DL-HASTE-5 mm (**B**), and DL-HASTE-3 mm (**C**). Lesion delineation was rated higher for DL-HASTE ((**B**,**C**), excellent image quality) compared with HASTE ((**A**), good image quality).

**Figure 7:**
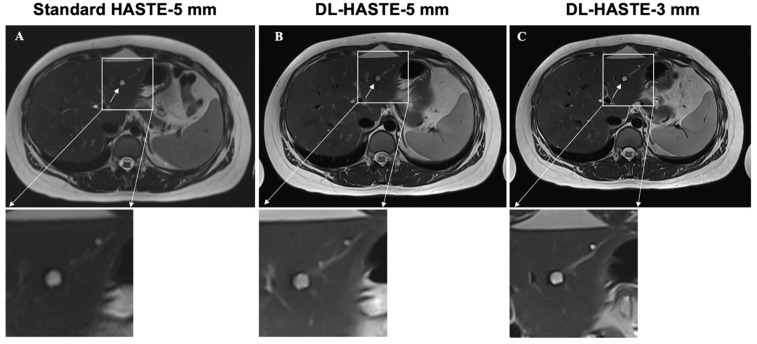
Delineation of cystic liver lesion on clinical standard-HASTE-5 mm (**A**), DL-HASTE-5 mm (**B**), and DL-HASTE-3 mm (**C**). Lesion delineation was rated higher for DL-HASTE ((**B**,**C**), excellent image quality) compared with HASTE ((**A**), good image quality).

Standard HASTE with a 5 mm slice thickness detected a total of 62 lesions (simple cysts, 43%; septated cysts, 5%; and solid masses, 52%) in 26 of the 30 study patients. In three of these patients, multiple lesions (*n* > 10) were detected. In these patients, only the largest lesion was included in the further analysis. Quantitative reading showed no significant differences concerning the number and maximum diameter (D_max_) of the detected lesions between the three sequences (standard-HASTE-5 mm, *n* = 62, D_max_ = 21.1 ± 18.1 mm, ICC: 0.995; DL-HASTE-5 mm: *n* = 62, D_max_ = 21.6 ± 18.4 mm, ICC: 0.997; DL-HASTE-3 mm: *n* = 62, D_max_ = 21.4 ± 18.1 mm, ICC: 0.99; standard-HASTE-5 mm vs. DL-HASTE-5 mm: *p* = 0.30; standard-HASTE-5 mm vs. DL-HASTE-3 mm: *p* = 0.08; DL-HASTE-5 mm vs. DL-HASTE-3 mm: *p* = 0.56). Lesion detectability was rated equally good for both DL-HASTE-5 mm and DL-HASTE-3 mm (5, IQR 4–5 vs. 5, IQR 4–5; *p* = 0.63) with significantly higher scores than those assigned to standard-HASTE-5 mm (4, IQR 4–5; *p* < 0.001). Examples of solid and cystic liver lesions are presented in Figure 6 and Figure 7. Further details are summarized in Table 3.

### 3.6. Acquisition Time

The mean acquisition time was 65 sec (three breath-holds) for clinical standard-HASTE-5 mm, 20 ± 2 s (range: 18–24 s; single breath-hold) for DL-HASTE-5 mm, and 24 ± 2 s (range: 22–26 s; single breath-hold) for DL-HASTE-3 mm. The acquisition times for DL-HASTE-5 mm and 3 mm were significantly shorter than for standard-HASTE-5 mm (*p* < 0.001). The acquisition time for DL-HASTE-3 mm was four seconds longer than for DL-HASTE-5 mm (range 3–5 s; *p* < 0.001) due to the higher number of slices acquired (45 vs. 35).

## 4. Discussion

In this perspective study, we assessed the feasibility of abdominal MRI with a deep-learning-reconstructed HASTE sequence (DL-HASTE) acquired with 3 and 5 mm slice thickness. By employing an in-line reconstruction technique instead of an off-line DL postprocessing reconstructed algorithm, we found that DL-HASTE provided robust and high-quality imaging within the routine clinical workflow. Quantitative and semiquantitative analyses demonstrated an improved image quality for DL-HASTE (*p* < 0.001) and a shorter acquisition time (DL-HASTE-3 mm, −63%; DL-HASTE-5 mm, −69%) compared with the routine clinical HASTE sequence. The image quality was further improved by reducing the slice thickness from 5 to 3 mm (*p* < 0.001), which did not compromise the SNR.

The technical feasibility of MRI sequences with DL reconstruction has already been demonstrated in several earlier studies [16,17,18,21,26]. Herrmann et al. were the first to describe the use of DL for HASTE imaging of the upper abdomen during a single breath-hold [15]. In their study and subsequent investigations, DL-HASTE with a 5 mm slice thickness and fat saturation (FS) provided better image quality than standard multi-breath-hold HASTE with FS. Our results confirm these findings for DL-HASTE-5 mm. In our study, we used a similar DL vendor reconstruction algorithm as Herrmann et al., which was trained offline with approximately 10,000 HASTE slices [15] acquired with 4–5 mm slice thicknesses. An important observation of our study is that the denoising algorithm also worked very well for HASTE with a 3 mm slice thickness. Thus, our results, for the first time, demonstrate the feasibility of 3 mm DL-HASTE at 3 Tesla. This is further support for the hypothesis that deep-learning-based reconstruction architectures with a physics-based data consistency generalize rather well across different applications and field strengths, likely because the neural-network-based image enhancement focuses on local correlations and denoising rather than the image content, as relevant for other deep learning applications. These DL algorithms do not need to be trained on identical MR sequences to work well in the clinical setting, a fact that may be related to the fidelity of k-space data in the employed network architecture. We chose a 3 mm slice thickness to achieve a reasonable anatomic coverage within a single breath-hold of less than 25 s. In our study, DL-HASTE-3 mm was acquired with 45 slices, resulting in 13.5 cm of coverage with a mean breath-hold time of 24 s. In a few patients, the breath-hold time was longer than 25 s because the TR had to be increased due to specific absorption rate (SAR) limitations. However, the longer breath-hold time rarely resulted in substantial motion artifacts due to the rapid single-shot acquisition scheme. Overall, DL-HASTE-3 mm achieved adequate anatomic coverage within a reasonable breath-hold time. Nevertheless, the anatomic coverage of DL-HASTE-3 mm was smaller than that of DL-HASTE-5 mm (17.5 cm). Therefore, for clinical practice, we see the value of DL-HASTE-3 mm as an additive sequence for the detailed assessment of small structures. Alternatively, DL-HASTE-3 mm could be performed as a multiple-breath acquisition for greater coverage. Finally, the introduction of new DL algorithms significantly reduced the examination time, mainly based on the reduction in the TA and the reconstruction time of 2–3 min after acquisition. This yields promising potential for MR workflow optimization.

To the best of our knowledge, one previous study has explored the feasibility of 3 mm T2w imaging of the upper abdomen with DL reconstruction [27]. The study was performed at 1.5 Tesla by Tajima et al. using multi-breath-hold fast advanced spin echo (FASE) and free-breathing fast spin echo (FSE). In accordance with our results, DL reconstruction improved the image quality of both FASE and free-breathing FSE of the upper abdomen. However, they did not compare their 3 mm sequences with standard 4–5 mm sequences. Hence, it is unclear whether their DL algorithm improved the image quality compared with the clinical standard. This is especially relevant, as their DL algorithm was only trained on knee and head MR sequences. Tajima et al. performed DL-FASE during multiple breath-holds with an acquisition time of 30 ± 4 sec for 40–80 slices. In contrast, we acquired 3 mm DL-HASTE during a single breath-hold to avoid composing artifacts in patients with a variable breathing depth. In accordance with our results, Tajima et al. see the benefit of 3 mm imaging, especially for the assessment of smaller structures like the pancreas and adrenal glands. A further study from Liu et al. [19] demonstrated that 4 mm single-breath-hold DL-HASTE demonstrated promising capabilities, providing significantly superior image quality and quantitative SNR for pancreatic lesions compared to standard T2-weighted sequences. In our study, the quantitative analysis using Laplacian variance as a sharpness metric provided a continuous scale and overcame the subjective selection bias associated with semi-quantitative analysis such as the Likert scale. Due to the quantitative analysis, our results showed that smaller anatomic structures showed better sharpness with a 3 mm compared to a 5 mm slice thickness, including the common hepatic and cystic duct, adrenals, portal vein, celiac trunk, splenic vessels, SMA, and intestinal wall. However, coarser structures, such as the edges of the kidneys, spleen, liver, and pancreas, or larger vessels, like the abdominal aorta, did not show a significantly improved image sharpness when ultra-thin 3 mm slices were acquired compared with a 5 mm slice thickness, resulting in a similar overall image quality for the two slice thicknesses in the semiquantitative analysis. Moreover, the evaluation of focal liver lesions showed no significant differences between the two slice thicknesses either. However, a DL-based 3 mm T2w sequence could be very helpful in the evaluation of complex cystic lesions in the pancreas and kidney due to the improved visualization of cystic septation and wall thickness, which often pose a challenge in everyday clinical practice [14,28,29]. Furthermore, a detailed pancreatic and bile duct analysis could provide a dedicated evaluation of small structures, such as side branches or neoplasms [30]. However, the primary focus of our study was to test the feasibility of DL-HASTE-3 mm and its impact on the delineation of small anatomic structures. Despite the many advantages of using DL algorithms, it is important to note that new non-physical artifacts are associated with them. In the current version, DL-HASTE occasionally showed tiny zebra-striped artifacts that did not interfere with the diagnostic image quality. They originated from interpolation, which was applied as a last step to the DL image and could be overcome by using a different interpolation algorithm in future versions of the DL-HASTE sequence. Further artifacts, such as banding artifacts related to Cartesian DL reconstruction or instabilities during the imaging reconstruction, which can potentially mask small pathologies, were reported [31,32].

This work is limited to the investigation of deep-learning-based improvements in image quality; there is already work on the complete or partial evaluation of image data using radiomics-based deep learning networks for image analysis. For example, the studies by Salvaggio G. et al. and Cairone L. et al. showed the radiomics-based analyzability of prostate lesions [33,34]. It would be interesting to investigate DL sequences in combination with these methods. Radiomics-based methods could possibly achieve better results in image interpretation based on a deep-learning-based enhanced image quality.

Despite our encouraging results, our study has some limitations. First, a relatively small number of participants from a single center were investigated. Second, in this initial study, we focused on overall anatomic details and quantitative analysis. Third, no reproducibility metrics were assessed in this study. Furthermore, there might be potential bias in the qualitative assessment related to familiarity with DL-HASTE images. However, considering the high image quality for the visualization of small structures, such as the cystic duct, we expect a particular benefit from DL-HASTE-3 mm for imaging cystic pathologies in future studies.

## 5. Conclusions

Single-breath-hold DL-HASTE with an ultra-thin 3 mm slice thickness is highly feasible for abdominal imaging and provides high-quality images while at the same time reducing the acquisition time by 63%. Ultra-thin 3 mm DL-HASTE imaging showed potential clinical advantages, particularly for the detailed evaluation of small anatomic structures, particularly ducts, vessels, and cysts. Future studies with larger numbers of cases are needed to detect the smallest pathological changes, such as complex cysts or pancreatic duct neoplasms.

## Figures and Tables

**Table 1:** Acquisition parameters of the three upper-abdomen T2w sequences.

Parameters	Standard-HASTE-5 mm	DL-HASTE-5 mm	DL-HASTE-3 mm
Orientation	Axial	Axial	Axial
TA, min:s	0:45	0:19 *	0:23 *
TE/TR, ms	95/1100	96/535 *	97/500 *
FA, degrees	160	130-90-110-130	130-90-110-130
iPAT	2	3	3
Matrix size	384 × 253	384 × 253	384 × 253
Motion Breathing regime (breath-hold)	3 With 10 s intervals	1	1
Slice thickness, mm	5	5	3
Slice	35	35	45
FOV ^◊^, mm^2^	308 × 379	308 × 379	308 × 379

HASTE, half-Fourier acquisition single-shot turbo spin echo; DL, deep learning; FA, flip angle; TA, time of acquisition; TE/TR, echo time/repetition time; iPAT, integrated parallel acquisition techniques; FOV, field of view. * TR was individually adjusted to specific absorption rate (SAR) limits, which resulted in variable TA. ^◊^ FOV in this table is the default value, which was individually adjusted clinically.

**Table 2:** Scoring criteria for qualitative image assessment.

Score	Overall Image Quality	Delineation of Abdominal Organ Margins	Sharpness of Hepatic, Bile, and Pancreatic Ducts	Conspicuity of renal Parenchyma, Renal Sinus, and Adrenal Glands	Conspicuity of Vessels	Respiratory Motion Artifact	Lesion Detection
5. Excellent	Excellent	Sharp and clear margin without blurring	Clearly delineated in all sections	Clearly depicted and delineated bilaterally	Sharp and clear margin without blurring	No artifacts	Clearly visible with sharp margins and inner structure
4. Good	Good	Clear margin with mild blurring	Clearly delineated in most sections, with only focal blurring	Clearly depicted with only focal blurring	Clear margin with only focal blurring	Visible but minor artifacts	Clearly visible with mild blurring
3. Moderate	Moderate but sufficient for diagnosis	Moderate blurring but sufficient for diagnosis	Moderate delineation in most of sections	Moderately depicted but sufficient for diagnosis	Moderate blurring but sufficient for diagnosis	Moderate artifacts but sufficient for diagnosis	Moderate blurring but sufficient for diagnosis
2. Poor	Poor and seriously affects diagnosis	Extreme blurring that affects diagnosis	Poor delineation in all sections	Apparent but unclear and affects diagnosis	Extreme blurring and compromising diagnostic evaluation	Poor and compromising diagnostic evaluation	Hardly visible
1. Unreadable	Unreadable	Unreadable	Invisible in all sections	Unreadable	Unreadable	Unreadable	Unreadable

**Table 3:** Image quality scores and *p*-value of DL sequences (DL-HASTE-5 mm, DL-HASTE-3 mm) compared with standard sequence (standard-HASTE-5 mm).

	Standard-HASTE-5 mm	DL-HASTE-5 mm	DL-HASTE-3 mm	Standard-HASTE-5 mm vs. DL-HASTE-5 mm	Standard-HASTE-5 mm vs. DL-HASTE-3 mm	DL-HAST-5 mm vs. DL-HASTE-3 mm
Overall image quality	4 (4–4)	5 (4–5)	5 (4–5)	<0.001 *	<0.001 *	0.527
Delineation of abdominal organ margins						
Overall sharpness Specific sharpness	4 (4–4)	5 (5–5)	5 (5–5)	<0.001 *	<0.001 *	<0.001 *
Liver	4 (4–5)	5 (5–5)	5 (5–5)	<0.001 *	<0.001 *	0.083
Spleen	4 (4–5)	5 (5–5)	5 (5–5)	<0.001 *	0.001 *	0.179
Kidney	4 (4–4)	5 (5–5)	5 (5–5)	<0.001 *	<0.001 *	0.046 *
Pancreas	4 (4–4)	5 (4–5)	5 (5–5)	<0.001 *	<0.001 *	0.005 *
Intestinal wall	4 (4–4)	5 (4–5)	5 (5–5)	<0.001 *	<0.001 *	<0.001 *
Sharpness of the ducts						
Common hepatic duct	4 (4–4)	4 (4–5)	5 (5–5)	<0.001 *	<0.001 *	<0.001 *
Cystic duct	3 (3–4)	4 (4–4)	5 (4–5)	<0.001 *	<0.001 *	<0.001 *
Common bile duct	4 (4–5)	5 (5–5)	5 (5–5)	<0.001 *	<0.001 *	0.003 *
Main pancreatic duct	4 (3–4)	5 (4–5)	5 (4–5)	<0.001 *	<0.001 *	0.003 *
Renal and adrenal conspicuity						
Adrenals	4 (3–4)	4 (4–5)	5 (4–5)	<0.001 *	<0.001 *	<0.001 *
Renal parenchyma	4 (4–4)	5 (5–5)	5 (5–5)	<0.001 *	<0.001 *	0.180
Renal sinus	4 (4–4)	5 (5–5)	5 (5–5)	<0.001 *	<0.001 *	1.000
Vessel conspicuity						
Abdominal aorta	4 (4–4)	5 (5–5)	5 (5–5)	<0.001 *	<0.001 *	0.157
Portal vein	4 (3–4)	5 (4–5)	5 (5–5)	<0.001 *	<0.001 *	<0.001 *
Celiac trunk	3 (3–4)	4 (4–5)	5 (4–5)	<0.001 *	<0.001 *	<0.001 *
Splenic vessel	3 (3–4)	4 (4–5)	5 (4–5)	<0.001 *	<0.001 *	<0.001 *
Renal vessel	4 (3–4)	4 (4–5)	5 (5–5)	<0.001 *	<0.001 *	<0.001 *
SMA	4 (3–4)	5 (5–5)	5 (5–5)	<0.001 *	<0.001 *	<0.001 *
Lesion detection	4 (3–4)	5 (4–5)	5 (5–5)	<0.001 *	<0.001 *	0.025 *
Respiratory motion artifacts	4 (4–5)	5 (4–5)	5 (4–5)	0.006 *	0.054	0.637

HASTE, half-Fourier acquisition single-shot turbo spin echo; DL, deep learning; SMA, superior mesenteric artery. * = statistically significant difference.

## Data Availability

The data presented in this study are available in this article.

## Abbreviations

DL	Deep learning
D_max_	Maximum diameter
FA	Flip angle
FS	Fat saturation
FOV	Field of view
HASTE	Half-Fourier acquisition single-shot turbo spin echo
IOA	Interobserver agreement
iPAT	Integrated parallel acquisition techniques
MRCP	Magnetic resonance cholangiopancreatography
NPV	Negative predictive value
PACS	Picture archiving and communication system
RTA	Respiratory triggered acquisition
SNR	Signal-to-noise ratio
SMA	Superior mesenteric artery
SPSS	Statistical Package for the Social Sciences (software)
T	Tesla
TA	Time of acquisition
TE	Echo time
TR	Repetition time
T1w	T1-weighted
T2w	T2-weighted

## References

[1] Semelka R.C., Kelekis N.L., Thomasson D., Brown M.A., Laub G.A. (1996). HASTE MR imaging: Description of technique and preliminary results in the abdomen. J. Magn. Reson. Imaging.

[2] Lewis C.E., Prato F.S., Drost D.J., Nicholson R.L. (1986). Comparison of respiratory triggering and gating techniques for the removal of respiratory artifacts in MR imaging. Radiology.

[3] Nanko S., Oshima H., Watanabe T., Sasaki S., Hara M., Shibamoto Y. (2009). Usefulness of the application of the BLADE technique to reduce motion artifacts on navigation-triggered prospective acquisition correction (PACE) T2-weighted MRI (T2WI) of the liver. J. Magn. Reson. Imaging.

[4] Griswold M.A., Jakob P.M., Heidemann R.M., Nittka M., Jellus V., Wang J., Kiefer B., Haase A. (2002). Generalized autocalibrating partially parallel acquisitions (GRAPPA). Magn. Reson. Med..

[5] Lee M.G., Jeong Y.K., Kim J.C., Kang E.M., Kim P.N., Auh Y.H., Chien D., Laub G. (2000). Fast T2-weighted liver MR imaging: Comparison among breath-hold turbo-spin-echo, HASTE, and inversion recovery (IR) HASTE sequences. Abdom. Imaging.

[6] Nakayama Y., Yamashita Y., Matsuno Y., Tang Y., Namimoto T., Kadota M., Mitsuzaki K., Abe Y., Katahira K., Arakawa A. (2001). Fast breath-hold T2-weighted MRI of the kidney by means of half-Fourier single-shot turbo spin echo: Comparison with high resolution turbo spin echo sequence. J. Comput. Assist. Tomogr..

[7] Hill D.V., Tirkes T. (2020). Advanced MR Imaging of the Pancreas. Magn. Reson. Imaging Clin. N. Am..

[8] Griffin N., Charles-Edwards G., Grant L.A. (2012). Magnetic resonance cholangiopancreatography: The ABC of MRCP. Insights Imaging.

[9] Chen F., Taviani V., Malkiel I., Cheng J.Y., Tamir J.I., Shaikh J., Chang S.T., Hardy C.J., Pauly J.M., Vasanawala S.S. (2018). Variable-Density Single-Shot Fast Spin-Echo MRI with Deep Learning Reconstruction by Using Variational Networks. Radiology.

[10] Wang X., Ma J., Bhosale P., Ibarra Rovira J.J., Qayyum A., Sun J., Bayram E., Szklaruk J. (2021). Novel deep learning-based noise reduction technique for prostate magnetic resonance imaging. Abdom. Radiol..

[11] Kim M., Kim H.S., Kim H.J., Park J.E., Park S.Y., Kim Y.H., Kim S.J., Lee J., Lebel M.R. (2021). Thin-Slice Pituitary MRI with Deep Learning-based Reconstruction: Diagnostic Performance in a Postoperative Setting. Radiology.

[12] Lee D.H., Park J.E., Nam Y.K., Lee J., Kim S., Kim Y.H., Kim H.S. (2021). Deep learning-based thin-section MRI reconstruction improves tumour detection and delineation in pre- and post-treatment pituitary adenoma. Sci. Rep..

[13] Tanabe M., Higashi M., Yonezawa T., Yamaguchi T., Iida E., Furukawa M., Okada M., Shinoda K., Ito K. (2021). Feasibility of high-resolution magnetic resonance imaging of the liver using deep learning reconstruction based on the deep learning denoising technique. Magn. Reson. Imaging.

[14] Morana G., Ciet P., Venturini S. (2021). Cystic pancreatic lesions: MR imaging findings and management. Insights Imaging.

[15] Herrmann J., Gassenmaier S., Nickel D., Arberet S., Afat S., Lingg A., Kündel M., Othman A.E. (2021). Diagnostic Confidence and Feasibility of a Deep Learning Accelerated HASTE Sequence of the Abdomen in a Single Breath-Hold. Investig. Radiol..

[16] Herrmann J., Wessling D., Nickel D., Arberet S., Almansour H., Afat C., Afat S., Gassenmaier S., Othman A.E. (2023). Comprehensive Clinical Evaluation of a Deep Learning-Accelerated, Single-Breath-Hold Abdominal HASTE at 1.5 T and 3 T. Acad. Radiol..

[17] Mulé S., Kharrat R., Zerbib P., Massire A., Nickel M.D., Ambarki K., Reizine E., Baranes L., Zegai B., Pigneur F. (2022). Fast T2-weighted liver MRI: Image quality and solid focal lesions conspicuity using a deep learning accelerated single breath-hold HASTE fat-suppressed sequence. Diagn. Interv. Imaging..

[18] Shanbhogue K., Tong A., Smereka P., Nickel D., Arberet S., Anthopolos R., Chandarana H. (2021). Accelerated single-shot T2-weighted fat-suppressed (FS) MRI of the liver with deep learning-based image reconstruction: Qualitative and quantitative comparison of image quality with conventional T2-weighted FS sequence. Eur. Radiol..

[19] Liu K., Li Q., Wang X., Fu C., Sun H., Chen C., Zeng M. (2024). Feasibility of deep learning-reconstructed thin-slice single-breath-hold HASTE for detecting pancreatic lesions: A comparison with two conventional T2-weighted imaging sequences. Res. Diagn. Interv. Imaging.

[20] Gassenmaier S., Warm V., Nickel D., Weiland E., Herrmann J., Almansour H., Wessling D., Afat S. (2023). Thin-Slice Prostate MRI Enabled by Deep Learning Image Reconstruction. Cancers.

[21] Tajima T., Akai H., Yasaka K., Kunimatsu A., Akahane M., Yoshioka N., Abe O., Ohtomo K., Kiryu S. (2022). Clinical feasibility of an abdominal thin-slice breath-hold single-shot fast spin echo sequence processed using a deep learning-based noise-reduction approach. Magn. Reson. Imaging.

[22] Herrmann J., Nickel D., Mugler J.P., Arberet S., Gassenmaier S., Afat S., Nikolaou K., Othman A.E. (2021). Development and Evaluation of Deep Learning-Accelerated Single-Breath-Hold Abdominal HASTE at 3 T Using Variable Refocusing Flip Angles. Investig. Radiol..

[23] Klein S., Staring M., Murphy K., Viergever M.A., Pluim J.P. (2010). elastix: A toolbox for intensity-based medical image registration. IEEE Trans. Med. Imaging.

[24] Pech-Pacheco J.L., Cristobal G., Chamorro-Martinez J., Fernandez-Valdivia J. Diatom autofocusing in brightfield microscopy: A comparative study. Proceedings of the 15th International Conference on Pattern Recognition. ICPR-2000.

[25] Shahryari M., Meyer T., Warmuth C., Herthum H., Bertalan G., Tzschätzsch H., Stencel L., Lukas S., Lilaj L., Braun J. (2021). Reduction of breathing artifacts in multifrequency magnetic resonance elastography of the abdomen. Magn. Reson. Med..

[26] Sheng R.F., Zheng L.Y., Jin K.P., Sun W., Liao S., Zeng M.S., Dai Y.M. (2021). Single-breath-hold T2WI liver MRI with deep learning-based reconstruction: A clinical feasibility study in comparison to conventional multi-breath-hold T2WI liver MRI. Magn. Reson. Imaging.

[27] Silverman S.G., Pedrosa I., Ellis J.H., Hindman N.M., Schieda N., Smith A.D., Remer E.M., Shinagare A.B., Curci N.E., Raman S.S. (2019). Bosniak Classification of Cystic Renal Masses, Version 2019: An Update Proposal and Needs Assessment. Radiology..

[28] Graumann O., Osther S.S., Karstoft J., Hørlyck A., Osther P.J. (2015). Bosniak classification system: Inter-observer and intra-observer agreement among experienced uroradiologists. Acta Radiol..

[29] Yenice M.G., Sam E., Arikan Y., Turkay R., Atar F.A., Sahin S., Incı E., Tuğcu V., Tasci A.I. (2020). Comparison of computed tomography and magnetic resonance imaging in the assessment of complex renal cysts by using the Bosniak classification. Actas Urol. Esp..

[30] Kim J.W., Park B.N., Nickel D., Paek M.Y., Lee C.H. (2024). Clinical feasibility of deep learning-accelerated single-shot turbo spin echo sequence with enhanced denoising for pancreas MRI at 3 Tesla. Eur. J. Radiol..

[31] Almansour H., Herrmann J., Gassenmaier S., Afat S., Jacoby J., Koerzdoerfer G., Nickel D., Mostapha M., Nadar M., Othman A.E. (2023). Deep learning reconstruction for accelerated spine MRI: Prospective analysis of interchangeability. Radiology.

[32] Antun V., Renna F., Poon C., Adcock B., Hansen A.C. (2020). On instabilities of deep learning in image reconstruction and the potential costs of, A.I. Proc. Natl. Acad. Sci. USA.

[33] Salvaggio G., Comelli A., Portoghese M., Cutaia G., Cannella R., Vernuccio F., Stefano A., Dispensa N., La Tona G., Salvaggio L. (2022). Deep Learning Network for Segmentation of the Prostate Gland With Median Lobe Enlargement in T2-weighted MR Images: Comparison With Manual Segmentation Method. Curr. Probl. Diagn. Radiol..

[34] Cairone L., Benfante V., Bignardi S., Marinozzi F., Yezzi A., Tuttolomondo A., Salvaggio G., Bini F., Comelli A., Mazzeo P.L., Frontoni E., Sclaroff S., Distante C. (2022). Robustness of Radiomics Features to Varying Segmentation Algorithms in Magnetic Resonance Images. Image Analysis and Processing. ICIAP 2022 Workshops. ICIAP 2022.

